# Evaluating the Three-Dimensional Printing Accuracy of Partial-Arch Models According to Outer Wall Thickness: An In Vitro Study

**DOI:** 10.3390/ma14226734

**Published:** 2021-11-09

**Authors:** Seung-Ho Shin, Jae-Sung Kwon, June-Sung Shim, Jong-Eun Kim

**Affiliations:** 1 Department of Prosthodontics, Oral Research Science Center, BK21 FOUR Project, Yonsei University College of Dentistry, Yonsei-ro 50-1, Seodaemun-gu, Seoul 03722, Korea; shin506@prostholabs.com; 2 Department and Research Institute of Dental Biomaterials and Bioengineering, Yonsei University College of Dentistry, Seoul 03722, Korea; jkwon@yuhs.ac; 3 Department of Prosthodontics, Yonsei University College of Dentistry, Yonsei-ro 50-1, Seodaemun-gu, Seoul 03722, Korea; jfshim@yuhs.ac

**Keywords:** 3D printing, CAD/CAM, additive manufacture, hollow model, internal structure, trueness

## Abstract

The printing accuracy of three-dimensional (3D) dental models using photopolymer resin affects dental diagnostic procedures and prostheses. The accuracy of research into the outer wall thickness and printing direction data for partial-arch model printing has been insufficient. This study analyzed the effects of wall thickness and printing direction accuracy. Anterior and posterior partial-arch models were designed with different outer wall thicknesses. After 3D printing, a trueness analysis was performed. Those with full-arch models were the control group. The full-arch model had an error value of 73.60 ± 2.61 µm (mean ± standard deviation). The error values for the partial-arch models with 1-, 2-, and 3-mm thick outer walls were 54.80 ± 5.34, 47.58 ± 7.59, and 42.25 ± 9.19 μm, respectively, and that for the fully filled model was 38.20 ± 4.63 μm. The printing accuracies differed significantly between 0 degrees and 60 degrees, at 49.54 ± 8.16 and 40.66 ± 6.80 μm, respectively (F = 153.121, *p* < 0.001). In conclusion, the trueness of the partial-arch model was better than that of the full-arch model, and models with thick outer walls at 60 degrees were highly accurate.

## 1. Introduction

Dental models are produced based on patient dental information to provide references for treatment such as counseling, diagnosis, and prosthesis production [1]. The recent rapid development of digital dentistry represented by computer-aided design (CAD)/computer-aided manufacturing has progressed digital dental procedures on oral cavities [2]. These improvements have helped the development of a more popular approach for digitizing patient oral information [3]. In digital dental procedures, the digital impression method using an intraoral scanner is widely used [4]. Digitalized oral patient information can be applied to software in a variety of ways, and combining this with a dental CAD program allows dentists and technicians to design the prosthesis faster [5,6]. In conventional dental procedures, error in the model affect prosthesis accuracy [7]. When using patient oral scan data to produce a dental model with three-dimensional (3D) printing technology, the most important aspect is to correctly implement real scan data [5]. In order to perform contour control and correct processing errors of the prosthesis before putting it in the oral cavity, the prosthesis must be evaluated using a model such as in a conventional dental procedure.

The major factors affecting the accuracy of 3D printing include mechanical characteristics such as the printing type and light intensity [8], and the printing direction, thickness, number of layers, and support structure configuration [9,10,11]. Various studies have also been conducted to determine the heterogeneity of different combinations of parameters, including the diversity of 3D printing materials [9,10,11,12,13,14,15]. A previous study evaluated the trueness of the full-arch dental model using various printing methods such as Fused Deposition Modeling (FDM), Stereo Lithography Apparatus (SLA), and Digital Light Processing (DLP) [8]. This evaluation indicated that the DLP printing method had the highest accuracy, followed by SLA and FDM. Printing direction was been considered to greatly influence the accuracy of the model when printing a full-arch dental restoration using SLA, with it being suggested that the model was accurate enough for clinical use when the direction is set after rotating 120 degrees [12]. In another study of the effect of layer thickness on model accuracy during printing, a printing layer thickness of 50 μm was the most accurate when using DLP printing, and thinner layers were more accurate when using SLA printing [11]. A volume comparison according to the presence of a cross-arch plate in a full-arch printed model and the design of its internal structure has been previously reported [16]. The present study evaluated how the presence of a cross-arch plate interacts with the internal structure, and evaluated the accuracy of the model according to its internal and support structures. Similar previous studies evaluated the presence of a cross-arch plate in a full-arch model and the printing stability, and conducted experiments based on printing variables, including the layer thickness during printing, and the printing method including FDM, SLA, and DLP. Those studies highlighted problems such as arch width reduction due to shrinkage in the posterior region [16].

For the convenience of patients and operators when manufacturing simple restorations including indirect restorations, such as inlays and onlays or single crowns, in the current conventional dental model manufacturing process, partial impressions are taken using a unilateral bite tray (partial tray) to produce a dental model [17,18]. During the oral scan procedure, partial scans are also preferred over full-arch scans in the prosthesis manufacturing process due to their higher accuracy [19] in terms of the scan itself and greater occlusion than traditional partial impressions [4,20]. It is therefore necessary to study the accuracy of manufacturing of both full-arch and partial-arch dental models using 3D printing.

Additive manufacturing of a dental model consumes a large amount of resin when the model has a large volume, like for the full-arch model. This leads to higher costs, and so clinicians and dental technicians are making various efforts to reduce the consumption of photocurable resin material [3]. In patient oral scan data, areas such as the palate or the tongue space can be printed after removing them in advance through modeling if they are unnecessary for the purpose of diagnosis or treatment. If necessary, the inside of the model can be empty, or the outer wall can be thin. This process has the advantage of reducing the amount of material used when developing the model [21,22]. Reducing the amount of resin used has the additional effect of reducing the time required for the 3D printing process [23,24].

Regarding the process of manufacturing a 3D-printed partial-arch model, research on differences in accuracy according to factors such as the internal structure, the degree of filling, and the outer wall thickness has been insufficient. This study therefore was designed to determine printing accuracy according to the outer wall thickness and printing direction of the anterior and posterior partial-arch models during the printing process. Previous research results designed the shells of the partial-arch models at thicknesses of 1, 2, 3, and 4 mm, and the empty and fully filled model groups were designated and evaluated. According to the purpose of the study, the null hypotheses were established as follows: (1) there will be no difference in print accuracy according to the outer wall thickness of the partial-arch models, (2) there will be no difference in the printing accuracy of the model according to the printing direction, and (3) there will be no difference in the printing accuracy of the model between the anterior and posterior regions.

## 2. Materials and Methods

**Figure 1:**
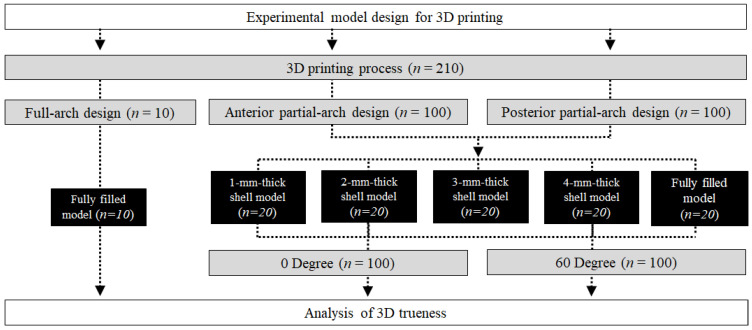
Flow chart of the study design.

The designs of the partial-arch model and overall experimental workflow of this study are presented in Figure 1.

To design the teeth models for each group, the maxillary tooth dentiform model (D85DP-500B.1, Nissin, Kyoto, Japan) was scanned using a tabletop scanner (Identica T500, Medit, Seoul, Korea). This tabletop scanner has <7-micron accuracy to ISO 12836, and phase-shifting optical triangulation, one arch per 12 s. The scan data was exported as a digital file in the Standard Triangulated Language (STL) format. The converted design file used modeling software (Meshmixer, Autodesk, San Rafael, CA, USA) to delete the palate region and other unnecessary structures and to design the overall full-arch shape.

**Figure 2:**
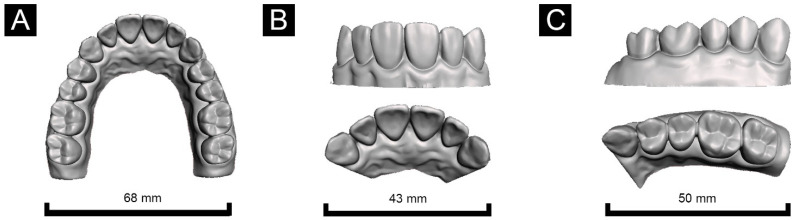
Designs of the (**A**) full-arch model, (**B**) anterior partial-arch model, and (**C**) posterior partial-arch model. Partial areas of the teeth and gingiva were designated and used to evaluate printing accuracy.

**Figure 3:**
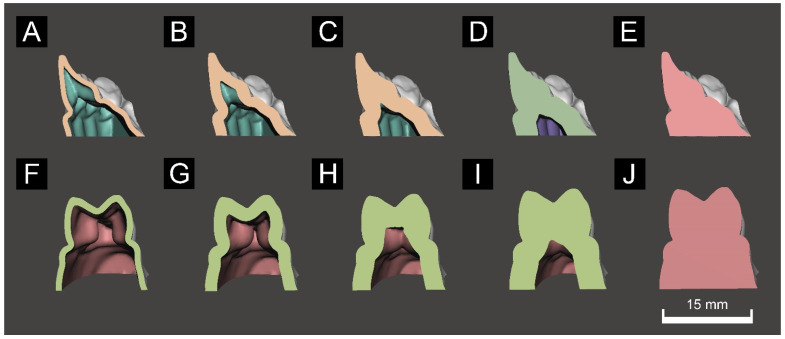
Cross sections of the 3D printed models. The anterior partial-arch groups consisted of a (**A**) 1-mm-thick shell model, (**B**) 2-mm-thick shell model, (**C**) 3-mm-thick shell model, (**D**) 4-mm-thick shell model, and (**E**) fully filled model. The posterior partial-arch groups consisted of a (**F**) 1-mm-thick shell model, (**G**) 2-mm-thick shell model, (**H**) 3-mm-thick shell model, (**I**) 4-mm-thick shell model, and (**J**) fully filled model.

Each partial-arch model was developed based on the full-arch model designed using 3D modeling software. An anterior partial-arch model (Group A) including the left canine tooth to the right canine tooth was designed, and a posterior partial-arch model (Group P) including the left canine tooth to the left second molar was designed (Figure 2). The hollow tool of Meshmixer was used to develop a hollow model with outer walls with thickness from 1 to 4 mm, and a fully filled model (Figure 3).

Photopolymer resin (Formlabs Gray resin, Formlabs, Somerville, MA, USA) and an SLA-type 3D printer (Form 3, Formlabs) were used to print the every models, and 10 printings were printed for each group. The layer thickness was set at 100 μm, and Groups A and P were printed in 0-degree- and 60-degree directions. The SLA-type 3D printer used for model printing has a point size of nm 85 µm, and the laser output is 50 mW, using Low Force Stereolithography printing strategy. For the control group, the full-arch group was printed at 0 degrees, and fully filled group was only one direction has printed. The model support structure of each group was attached below the model.

Table 1 lists the resin consumption according to the type of printed tooth model.

All denture bases were washed at 20 °C and 50% humidity and 90% isopropyl alcohol using a wash machine (Twin Tornado, Medifive, Incheon, Korea), and polymerized according to the manufacturer’s instructions using a UV polymerization unit (CureM D102, Sona Global Company, Seoul, Korea). The postcuring device covered the spectral range of 385–405 nm using a 72-W Ultraviolet Light Emitting Diode. The scan data of the printed model obtained using a tabletop 3D scanner were saved as STL files and imported into a 3D inspection software program (Geomagic Control X, 3D Systems, Rock Hill, SC, USA). Only each tooth area and the surrounding gingival area were identically designated for comparisons, and the scan data of each group were superimposed on the outline of the denture base design reference data, and trueness was calculated after performing complete alignment.

The best-fit process was first performed while sorting scan data for trueness. The best-fit process calculated and registered the mesh point deviations of the loaded 3D model based on the reference model, then evaluated the mesh set deviation based on the calculated deviation at each point and the dependence on the variables, and then determined the optimal steps to proceed with fit alignment [25]. The comparison of the trueness values of the 3D models refers to evaluation of the mesh point deviation based on the reference model after the digital scan file underwent the best-fit process alongside the reference model to verify the volumetric accuracy of the printed model [26]. The tooth area and part of the gingiva of the prespecified reference data were then selected, and the scan data of all models were compared based on the selected area to determine trueness. Trueness values were calculated using the surface deviation data and root–mean–square error (RMSE). RMSE is calculated as the square root of the mean square by which an error indicator can be identified in units similar to the actual value, which is then applied to measure the volume and shape variability of a surface [27]. In this study, the RMSE could therefore be judged as the error value of trueness, and was calculated using the following formula:

(1) $$\mathrm{RMSE}\;=\frac{1}{\sqrt{n}}\cdot \sqrt{\overset{n}{\underset{i=1}{\sum }}{\left(x_{1,i}-x_{2,i}\right)}^{2}}$$

where $x_{1}$ and $x_{2}$ are the predicted and actual values, respectively, and  i is the starting term that determines the sum of the formula; since it starts from the first term,  i=1 is a fixed value. Statistical analysis was performed by determining the RMSE values using the above formula. RMSE values further from 0 indicated increasing error relative to the reference model and hence lower volume stability, and vice versa. A high RMSE therefore represents a large error relative to the reference model. The RMSE values for all scan data were statistically analyzed to compare the trueness values between groups, and the average RMSE value of each group was used during the statistical analysis.

Statistical analysis was applied to the results for each group using standard statistical software (SPSS version 25.0). All acquired data were subjected to Levene’s test to determine homoscedasticity and the Shapiro–Wilk normality test to determine normality. Three-way ANOVA was conducted to determine differences between the outer wall thickness and printing direction in each anterior partial shape and posterior partial shape of each group. One-way ANOVA was used to compare differences over time after printing for the same exterior wall thickness group. Bonferroni correction was used as a post-hoc test. The significance cutoff of all tests was set at α = 0.05.

## 3. Results

**Figure 4:**
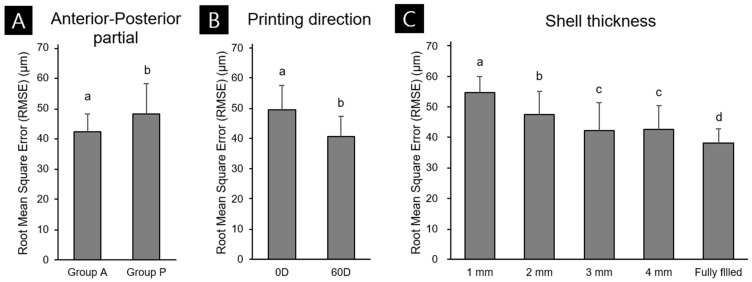
RMSE values from three-way ANOVAs for Groups A and P (**A**), printing direction (**B**), and (**C**) outer wall thickness. Lower-case letters indicate significant differences (*p* < 0.05). Data are mean and standard deviation values.

Figure 3 shows the results of the three-way ANOVA trueness analysis according to differences in outer wall thickness, printing direction, and partial shape. The mean deviations for Groups A and P were 42.21 ± 5.94 and 47.99 ± 10.14 μm (mean ± standard deviation), respectively, and thinner outer walls had lower printing accuracy for partial-arch models and higher printing accuracy for the fully filled model (Figure 4C). In the partial-arch model, the anterior shape was more accurate than the posterior shape (Figure 4A). The deviation was 49.54 ± 8.16 μm when printing at 0 degrees and 40.66 ± 6.80 μm when printing at 60 degrees, indicating that the 60-degree group had a lower accuracy error (Figure 4B). Partial shape and printing direction were significantly related (F = 63.15, *p* < 0.001), whereas printing direction and outer wall thickness did not (F = 2.16, *p* = 0.75). The shape of the partial-arch model and outer wall thickness did not have a significant effect (F = 2.05, *p* = 0.089). Partial shape, printing direction, and the interaction of the three outer wall thickness factors had a significant relationship (F = 3.1, *p* = 0.017).

**Figure 5:**
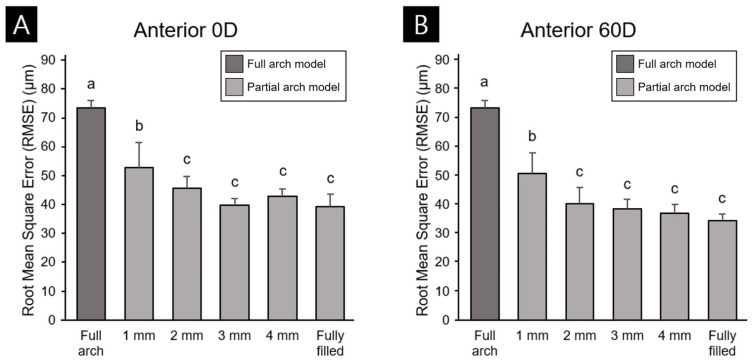

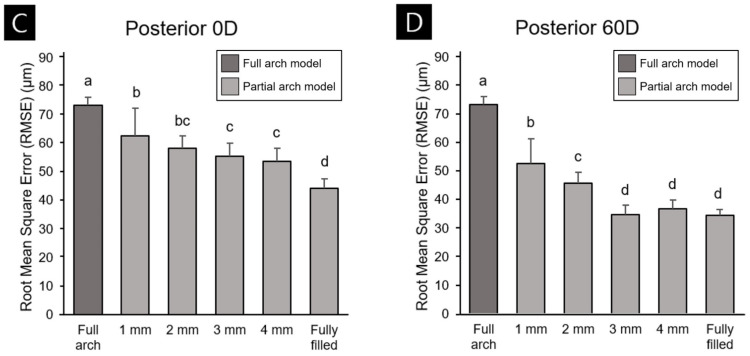
RMSE values in the anterior 0-degree group (**A**), anterior 60-degree group (**B**), posterior 0-degree group (**C**), and posterior 60-degree group (**D**), Lower-case letters indicate significant differences (*p* < 0.05). Data are mean and standard deviation values.

Comparisons of RMSE values representing the trueness of the anterior (Figure 5A,B) and posterior (Figure 5C,D) partial-arch models indicated lower error values than for the full-arch model, which had an error value of 73.6 ± 2.60 µm across all groups. Within the partial-arch group, the largest errors were indicated in the 0-degree and 60-degree anterior and posterior partial-arch groups: 52.8 ± 8.7, 50.9 ± 7.1, 62.7 ± 9.7, and 52.8 ± 8.7 µm for the anterior 0-degree 1-mm, anterior 60-degree 1-mm, posterior 0-degree 1-mm, and posterior 60-degree 1-mm groups, respectively. This suggests that accuracy error decreases as the outer wall becomes thicker, until the model is completely filled. In the fully filled group, the error values for the anterior 0-degree and 60-degree groups were 39.3 ± 4.4 and 34.6 ± 2.1 µm, respectively, and those of the posterior 0-degree and 60-degree groups were 44.3 ± 3.3 and 34.6 ± 2.1 µm, respectively. The comparison between printing directions also indicated that the errors for both the anterior (Figure 5C) and posterior (Figure 5D) models were lower for 60 degrees than for 0 degrees.

**Figure 6:**
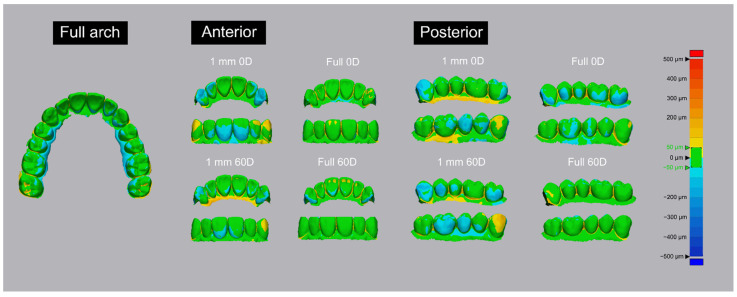
Scan color maps for each printing direction and outer wall thickness of the full-arch and anterior and posterior partial-arch groups. Comparisons with the full-arch group revealed that the anterior and posterior 0 degree partial-arch groups had large volume changes. For the 1-mm-thickness groups, the fully filled model and anterior and posterior partial-arch groups printed at 60 degree were compared, revealing relatively small volume changes.

The 3D color map analysis results in Figure 6 indicate the volumetric deformations for different outer wall thicknesses in the full-arch, anterior-arch, and posterior-arch groups. A different type of volumetric deformation was observed between the full-arch and partial-arch groups, and in the full-arch group, with the posterior part mostly contracting to the lingual side. In the partial-arch group, the 0-degree and 60-degree 1-mm groups and the fully filled anterior and posterior partial-arch groups had relatively large differences in volume, allowing the changes to be clearly visualized. In both the anterior and posterior partial-arch groups, the volume difference between the 1-mm and full-arch groups was noticeable. In the anterior 0-degree group, contractions mostly occurred in the canine and anterior buccal parts. In the anterior 60-degree group, a volume error of the area where the support structure was attached was confirmed. In the posterior partial-arch group, a large volume difference was found according to both the printing direction and outer wall thickness, and the contraction was indicated to have occurred mostly in the margin area of the model and in the distal direction of the canine and posterior teeth, corresponding to the two ends of the model. In the posterior 0-degree 1-mm group, these features were very prominent, and the tooth margin had a prominent shape deformation, whereas in the posterior 60-degree 1-mm group, the volumes of the teeth were similar but the shape deformation of the margin was greatly reduced. The posterior full 0-degree and 60-degree groups indicated a good volume condition.

## 4. Discussion

This study evaluated whether the volumetric accuracy of a model is affected when the outer wall thickness of the model differs during the design process or when the angle is set differently during printing, with the aim of identifying factors that can minimize volume changes during 3D printing of a partial-arch model The results of this study indicated that the full-arch group had a higher accuracy error than the partial-arch group, and that the accuracy error during 3D printing decreased as the outer wall of the partial-arch group became thicker. It was also found that in the partial-arch group, the posterior shape had a higher accuracy than the anterior shape. These findings indicate that there are significant variations in printing accuracy according to outer wall thickness, printing direction, and whether the model has an anterior or posterior shape. This caused us to reject the first null hypothesis of there being no difference in accuracy depending on the outer wall thickness of the partial-arch group, the second null hypothesis of the printing direction angle having no effect, and the third null hypothesis of there being no difference in the printing accuracy of the model according to an anterior or a posterior shape.

In the past, several studies measuring the error of the plaster model and the 3D printing model have measured the precision of the model by point-to-point measurement between the cusp and the cusp [28,29]. Since this method is a 2D linear measurement, there is a disadvantage that the overall error of the model cannot be analyzed. In addition, a study conducted with 2D linear measurement cannot be evaluated in the same way as the error value of 3D measurement including RMSE evaluation. Therefore, the 3D scan measurement method was applied in this study. In a review of various 3D model printing studies by Etemad-Shahidi et al. [30], the trueness of the SLA-type 3D printing process was confirmed to have an error measurement value of <100 µm. This is consistent with <100 µm set as a clinically applicable error range in a previous study [7,9,31]. The error values generated in our study are within this clinically applicable error range. This study applied an SLA-type 3D printing process. The printing accuracies of groups with outer wall thicknesses of 1–4 mm and fully filled groups were evaluated, which revealed that the accuracy increased as the outer wall thickness increased regardless of the printing direction. The anterior partial-arch group had a higher overall accuracy error than the posterior partial-arch group. The fully filled group had greater accuracy than the partial-arch models. Both the 3D printing and postcuring processes of the model polymerize the photopolymer resin, and the shrinkage during this process has been reported to affect the accuracy of produced objects [32,33]. Our color map analysis of the overlap between the reference and scan data revealed volume changes in both the maxilla and mandible that appeared to be related to shrinkage during polymerization [34,35]. As the thickness of the model increases, it stabilizes the model itself, which is thought to resist the overall deformation during the shrinkage process following postcuring [16]. As the thickness of the model decreases, its structure diminishes, and volumetric deformation increases. Similar results were obtained by Rungrojwittayakul et al. [36], who reported that thinner outer walls in a 3D print have lower stability. The present study evaluated the differences trueness and precision between groups by designing a model in which the outer wall thickness of the full-arch model was 2-mm and a fully filled model. A model with a 2-mm-thick outer wall printed using continuous liquid interface production had an error of 45 μm, and the solid/fully filled model had a mean error of 35 μm. The 2-mm-thick outer wall model printed using the DLP 3D printer had a mean error of 77 μm, as did the solid model. This study also found that the 1-mm group (the thinnest outer wall) was the least accurate regardless of printing direction. A similar result was reported by Shin et al. [16]. A full-arch model replaced the tongue and palatal space with a cross-arch plate, and thickness of the outer wall and internal structure of the full-arch model were designed in a U shape, and the differences in volumes were compared in the present study. The fully filled cross-arch plate model had the lowest RMSE value, and in the U-shaped model, the fully filled model also had the lowest RMSE value. In the present study, the U-shaped model without a support structure had both molars contracting to the lingual side. These values tended to be larger when there was no internal structure, or the outer wall of the model was more thin.

This study evaluated the volume differences of printed models according to the printing direction. Comparing the trueness of the group in which the base was printed at 0 degrees parallel to the printer bed and the group in which the model was tilted 60 degrees in the lingual direction indicated that the print accuracy was generally greater in the 60-degree group. Previous experiments that involved 3D printing of structures such as crowns and denture bases indicated that the volumetric accuracy of the print was better when 3D printing was performed at an angle [9,12]. Jin et al. [9] studied the effect of the printing direction angle for denture bases, which had a volume similar to that of their full-arch model. As the angle increased, the maxillary arch gradually deviated from the palate to the posterior suture of the palate, and in the mandibular arch, the edentulous labial angle to the ridge crest expanded. The maxillary and mandibular RMSE values at 90 degrees were 0.095 and 0.114 mm, respectively, and those at 100 degrees were 0.079 and 0.103 mm, respectively. The maxillary and mandibular RMSE values at 135 degrees were 0.087 and 0.123 mm, respectively, and those at 150 degrees were 0.088 and 0.136 mm, respectively. That study therefore indicated that the printing accuracy was good when the denture base was printed at angles of 100–135 degree. Crown bridges are very small, printed models, which have a volume similar to the partial-arch model rather than to the full-arch model. Tooth bridge printing studies have therefore been conducted, such as that by Park et al. [37] that compared the printing accuracy for each angle of a crown bridge. In that study, the crown was rotated at 0, 30, 45, 60, and 90 degrees, and the printing was then performed to the abutment, and the internal gap volume of the print was then measured using micro-CT.

In the present study, the volume of each internal gap was evaluated; the smallest volume was 45.5 ± 2.5 µm in the 90-degree 100-µm group, and the largest was 53.7 ± 2.6 µm in the 0-degree 100-µm group. For a 50-μm layer thickness, the 60-degree models were the smallest, followed by the 45-degree models. When considering the internal gap volumes obtained in this study together, printing directions of 45 degrees and 60 degrees were recommended, and the margin fit of a 3D-printed prosthesis with a 100-µm-thick layer during printing was similar to that for a 50-µm-thick layer. These research results can be mostly attributed to the printer bed being affected by the *Z*-axis during the 3D printing process [38]. The *Z*-axis is not a continuous variable in the 3D printing process, unlike the X-axe and Y-axe, but instead varies with the layer thickness. Therefore, all 3D printed models are directly affected by mechanical factors such as layer thickness [11] and 3D printing settings during the lamination process. Regarding DLP- and SLA-type printers, the tension that causes the separation from the bottom film of the printer resin tank is also induced during the printing process, and tension at the time of separation can cause a discrepancy in the lamination process for each layer [39,40]. Similarly, Alharbi et al. [12] found that the printing direction greatly affected the accuracy of SLA printing in full-coverage dental restorations. That study indicated optimal clinical application suitability when printing at an angle of 120 degrees from the forward direction toward the centrifugal direction. In that study, after 3D printing at angles of 90, 120, 135, 150, 180, 210, 225, 240, and 270 degrees relative to the crown, the lowest RMSE values were in the models rotated by 90 degrees and 120 degrees with a thin support thickness, at 0.027 and 0.029 mm, respectively. When printing at an angle of 120 degree, we could not find the fatal error of the margin part due to its support structure attachment. The results indicated that the printing result was better than when it was performed at 90 degrees. These findings together suggest that the volume of the printing result is good when printing a model at an angle.

In clinical practice, dental models greatly affect the fit or adaptation when manufacturing crowns and other prostheses. In particular, when manufacturing a model using 3D printing, the accuracy of the printed object can be affected by distortion during the postcuring or printing process, unlike when manufacturing using a conventional model. 3D printing using photopolymer resin requires considerable control during the printing process, and the outer wall thickness and printing direction are factors that directly affect the printing process. Partial-arch models are also not free from volume changes according to these factors, but as indicated in this study, the volume was stable as the outer wall became thicker and the model was tilted to a certain angle. Therefore, when printing a partial-arch model after filling the inside and at a direction of 60 degrees, the print was more stable, even compared with a full-arch model.

This study had several limitations. Because its focus was on the print accuracy according to outer wall thickness and print angle, the analysis was carried out using one type of 3D printer and one type of photopolymer resin. When subsequently printing a partial-arch model, other factors can affect the printing accuracy according to the mechanical properties of 3D printing, including the thickness of the laminated layer, the type of photopolymer resin, and the type of 3D printer. When different volumetric deformations accumulate depending on the shape of the palatal arch, this may also affect the occlusion between the upper and lower palatal arch models. It is therefore thought that a wider range of research results need to be obtained when using models with various partial-arch designs.

## 5. Conclusions

The following conclusions can be drawn from the results obtained in this study:

Trueness values in the anterior and posterior partial-arch groups indicated that the accuracy increased with the outer wall thickness, and that the fully filled model was the most accurate. The anterior and posterior partial-arch groups exhibited larger errors for tilting at 60 degrees in the 1- and 2-mm external-wall-thickness groups, compared with 0 degrees. However, the 3-mm, 4-mm, and fully filled groups exhibited lower errors when printed at 60 degrees. The anterior partial-arch group had lower printing accuracy than the posterior partial-arch group, which appears to be due to the volume differences varying with the size of the model.

Through this study, it was found that it is more advantageous to produce a partial-arch model rather than a full-arch model when printing and utilizing a 3D printed model in clinical practice. In addition, it is thought that it would be better not to empty the inside in the work that requires more accuracy.

## Figures and Tables

**Table 1:** Resin consumption according to model design (unit: mL).

Model Name	Anterior Partial-Arch Model (Group A)	Posterior Partial-Arch Model (Group P)
Full-arch fully filled model	16.28	
1-mm-thick shell model	4.38	5.23
2-mm-thick shell model	5.68	6.70
3-mm-thick shell model	6.88	7.84
4-mm-thick shell model	7.33	8.62
Fully filled model	7.76	9.64

## Data Availability

Not applicable.
